# Workplace Mental Health and Social Media Addictive Behaviours: The Application of Mattering Theory

**DOI:** 10.1177/08445621261428242

**Published:** 2026-03-02

**Authors:** Tsorng-Yeh Lee, Chang Su, Gordon L. Flett

**Affiliations:** 1 School of Nursing, Faculty of Health, 7991 York University, Toronto, Ontario, Canada; 2 Department of Psychology, Faculty of Science, 1916 Brandon University, Brandon, Manitoba, Canada; 3 Department of Psychology, Faculty of Health, 7991 York University, Toronto, Ontario, Canada

**Keywords:** Mattering, anti-mattering, social media, job satisfaction, COVID-19

## Abstract

**Purpose:**

The present study examined associations between adjustment and job satisfaction and multiple dimensions of mattering—including general mattering, mattering at work, anti-mattering experiences, and fears of not mattering—among working adults. A secondary, exploratory aim was to examine whether mattering-related outcomes differed as a function of personal COVID-19 infection history.

**Method:**

A cross-sectional study was conducted with 60 employees working primarily from home during the COVID-19 pandemic. Participants completed self-report measures assessing mattering, well-being, depression, social media addiction, and job satisfaction.

**Results:**

General mattering was positively associated with work mattering and affective job satisfaction, whereas anti-mattering was negatively associated with work mattering, well-being, and affective job satisfaction. Well-being was positively related to both work mattering and affective job satisfaction, supporting the protective role of perceived significance in the workplace. Social media addiction was positively correlated with non-mattering and depression, suggesting that diminished perceptions of mattering may be linked to maladaptive online behaviors and poorer mental health. Exploratory independent-samples t-tests indicated that individuals with a history of COVID-19 infection reported lower general mattering, work mattering, and affective job satisfaction, and higher anti-mattering, compared with those without infection.

**Conclusion:**

The findings highlight the importance of mattering as a resource for employee well-being and job satisfaction. Experiences of anti-mattering were associated with poorer mental health and greater vulnerability to problematic social media use. Exploratory differences linked to COVID-19 infection history suggest pandemic-related stressors may negatively influence employees’ sense of value and satisfaction. These results underscore the need for organizational practices that foster employees’ sense of mattering, particularly during periods of disruption.

## Background & Purpose

Workplace Mental health and well-being are essential to employees (Sarkar et al., 2024). It can be influenced by the work environment, characterized by inclusion, respect, equity, and a supportive culture. Mental Health is defined by the World Health Organization (WHO, 2025) as “a state of well-being in which an individual realizes his or her abilities, can cope with the normal stresses of life, can work productively and can contribute to his or her community.” Employees who experience better mental health and well-being within the workplace, shaped by psychosocial working conditions such as job demands, organizational support, and work environment, are better able to perform effectively, manage work-related challenges, and maintain productivity (Government of Canada, 2025). In contrast, poor workplace mental health and reduced well-being, often arising from adverse work conditions, are associated with lower job satisfaction, impaired performance, increased absenteeism, and a heightened risk of exhaustion and burnout (de Oliveira et al., 2023).

According to a recent global survey conducted across 11 countries involving 4,000 employees, managers, and business leaders, 63% of employees believe their managers or employers have ignored their voices (Workplace Intelligence, 2025). *Employee mattering* refers to employees’ perceptions that they are valued, noticed, and that their contributions are important within the organization (Flett & Zangeneh, 2020). One key manifestation of employee mattering is the extent to which employees feel their **voices** are heard and considered in workplace decisions, signalling recognition and respect from leadership and peers. When employees experience low levels of mattering, they are more likely to report disengagement, reduced well-being, and diminished motivation, all of which can negatively affect organizational functioning (Reece et al., 2021). Despite its relevance to organizational health and employee success, research examining employee mattering in workplace contexts remains limited, highlighting the need for further investigation (Flett & Zangeneh, 2020; Reece et al., 2021). Workplace mental health is a significant concern for employers, and these findings highlight the need for better support and attention to employees’ voices and well-being.

Workplace mental health has increasingly been recognized as a critical determinant of employee well-being and organizational functioning. To guide organizations in promoting psychologically healthy work environments, the U.S. Surgeon General outlined five core themes for workplace mental health, including protection from harm, connection and community, work–life harmony, organizational trust, and mattering at work (Rosemberg & McPhaul, 2023). Among these themes, *mattering at work*—employees’ perceptions that they are valued, recognized, and that their contributions are meaningful—has received comparatively less empirical attention, despite its central role in shaping engagement, motivation, and well-being. Accordingly, the present study focuses specifically on mattering at work to address this gap and to better understand its implications for workplace mental health.

Dr. Murthy further suggests that organizations can foster a healthier work culture by implementing specific practices that support *mattering at work*, such as providing a living wage, involving employees in workplace decision-making, connecting individual roles to the broader organizational mission, and cultivating a culture of gratitude and recognition (ASH Media, 2025).

Drawing on theory and research highlighting mattering as a central psychosocial construct, the current study examined associations between multiple dimensions of mattering and indicators of psychological and work-related functioning. Adjustment was included as outcome given evidence that perceptions of mattering are closely tied to emotional well-being, stress, and individuals’ ability to cope with environmental demands. Job satisfaction was examined as a key occupational outcome, as feeling valued and significant in one's work role is theorized to be foundational to positive work attitudes and engagement.

In addition to positive forms of mattering, this study also focused on mattering vulnerability, including anti-mattering feelings and fears of not mattering. These facets reflect concerns about invisibility, insignificance, and exclusion, which may be especially salient during periods of social disruption and uncertainty. Despite growing interest in mattering, relatively little research has simultaneously examined both positive and negative dimensions of the construct in relation to adjustment and job satisfaction within applied work contexts.

Accordingly, the purpose of the present study was to examine the associations between adjustment and job satisfaction and multiple dimensions of mattering—including general mattering, mattering at work, anti-mattering experiences, and fears of not mattering—among working adults. In addition to examining mattering-related constructs, it is important to account for the broader pandemic-related stressors present during the period of data collection. The COVID-19 pandemic disrupted work routines, intensified uncertainty, and increased reliance on remote work and digital communication, all of which may influence employees’ perceptions of being valued, their psychological well-being, and their work experiences. Individuals who experienced COVID-19 infection may have faced additional stressors, including health-related anxiety, recovery demands, and social disruption, which could further shape perceptions of mattering and job satisfaction. Accordingly, the present study considered pandemic impact as a contextual factor and explored differences in mattering-related outcomes based on personal COVID-19 infection history.

## Mattering

Prilleltensky and colleagues (Prilleltensky & Prilleltensky, 2021) define mattering as involving two complementary psychological experiences: being feeling valued by others (e.g., noticed, important, appreciated) and adding value (making a meaningful contribution to others and one's social context). Individuals know they matter when they feel noticed, valued, and needed others (externally), it also comes from within by finding individual values and making positive impacts (Prilleltensky, 2020). Mattering has been recognized as a crucial psychological resource that helps people cope with and adjust to new environments (Flett, 2019). It is highly relevant in the workplace, as low levels of perceived mattering are linked to higher burnout, increased turnover, reduced productivity, and poorer mental health outcomes, whereas higher perceptions of mattering function as a protective factor. Employees who feel valued and believe that they matter at work are less vulnerable to emotional exhaustion and psychological distress, and are therefore more resilient during challenging times. This heightened sense of self fuels adaptability and resilience (Flett, 2019; Flett, 2022). The feelings of mattering indicate that individuals have a fundamental need to feel significant. Mattering serves as a protective factor and contributes to well-being (Flett, 2019). It is highly relevant in the workplace, affecting important job-related indicators such as burnout, turnover, productivity, and mental health issues. Employees who feel valued and that they matter at work are more resilient during challenging times. This heightened sense of self fuels adaptability and resilience (Flett, 2019; Flett, 2022). Studies have shown that mattering can predict outcomes related to job satisfaction, stress, depression, and well-being (Flett, 2022; Tonini et al., 2025). In addition, fewer studies have examined the relationship between mattering and employees’ demographic factors, such as education and family income. This gap is important because education and income are closely tied to power, opportunity, and access to workplace resources, which may shape employees’ perceptions of being valued and significant at work. Understanding these differences can help identify groups for whom mattering may be especially vulnerable.

The current study has multiple interrelated goals that reflect the complexity of the mattering construct. Measuring how much people feel they matter to others in general and in the workplace is important. Recent studies have shown that feelings of not mattering, as measured by the Anti-Mattering Scale, are not simply the opposite of positive feelings of mattering. This is likely due to distinct feelings and motivational orientations that come with feeling valued and essential versus feeling unseen, unheard, and unimportant (Flett et al., 2022).

The construct of *mattering* has been increasingly recognized as a fundamental psychological need, reflecting individuals’ perceptions that they are significant, valued, and important to others (Flett, 2022; Flett et al., 2025). Research across educational, social, and clinical contexts has consistently linked positive feelings of mattering to well-being and adjustment, underscoring its relevance for understanding psychosocial functioning.

Despite this growing theoretical and empirical literature, relatively little research has examined mattering within workplace contexts or its associations with work-related outcomes. In particular, research linking mattering to job satisfaction remains limited, with most existing studies relying on narrow or global indicators of the construct (Connolly & Myers, 2003; Flett, 2018; Rayle, 2006; Richards et al., 2020).

This gap is notable given that mattering is a multidimensional construct and may operate through multiple pathways in organizational settings. Accordingly, the present study incorporates multiple indices of mattering to provide a more comprehensive assessment of how feeling valued and significant at work relates to employees’ job satisfaction.

The purpose of the current study was therefore to extend prior research by examining the associations between multiple dimensions of mattering and job satisfaction, thereby advancing understanding of mattering as a meaningful predictor of work-related well-being.

The present study is grounded in contemporary theory and research on mattering, which conceptualizes mattering as a multidimensional construct with important implications for individual well-being and functioning (Flett, 2022; Flett et al., 2025). Within workplace contexts, existing research has consistently linked positive perceptions of mattering to favorable outcomes, including job satisfaction; however, this body of work remains relatively limited in scope and depth (Flett, 2018).

In particular, prior studies have tended to rely on global or narrow indicators of mattering, leaving unanswered questions about how different dimensions of mattering relate to work-related outcomes. As a result, the extent to which a more comprehensive assessment of mattering may extend existing findings remains unclear (Connolly & Myers, 2003; Rayle, 2006; Richards et al., 2020).

To address these gaps, the current study includes multiple indices designed to capture the broader mattering construct. Accordingly, it was expected that the resulting pattern of findings would extend past prior research linking general perceptions of mattering with job satisfaction by offering a more nuanced understanding of how mattering operates in the workplace.

## Workplace Mental Health

It has been more than five years since the World Health Organization (WHO) declared the COVID-19 pandemic on March 11, 2020. According to the WHO's recent report (2024), COVID-19 positive cases were 6.3% across 80 countries during the week ending on 26 May 2024. Globally, 94 countries reported COVID-19 cases, and 28 countries reported COVID-19 deaths. This does not reflect the number of countries where cases or deaths occur, as many countries have stopped or changed the reporting frequency. Although vaccination and new drug inventions appear to be controlling the further spread of infection, recent cases in Canada and other countries worldwide are making people realize that the pandemic may not be over yet. Working from home has become a welcome strategy since the COVID-19 pandemic.

The COVID-19 pandemic accelerated the adoption of remote work. Many employees appreciate the idea of working from home (Su et al., 2022). Studies have highlighted several challenges, especially for those with school-age children. These employees not only have to meet work demands but also help their children with their learning.

The recent easing of public health restrictions has led many companies to mandate that employees return to the office, while others are opting for a hybrid model that combines remote work with in-office time. Conversely, certain companies have decided to make remote work a permanent arrangement. According to a survey by the Harris Poll, almost 90% of employees have expressed concerns about returning to the office, citing worries about physical safety due to the highly contagious variants, as well as the challenge of readjusting their interactions with colleagues (Velasquez, 2021). Not to mention the financial and health costs of commuting after returning to the office.

During the COVID-19 pandemic, many organizations reported short-term increases in employee productivity; however, these gains often coincided with heightened anxiety, burnout, and psychological strain among employees. Emerging evidence suggests that productivity increases achieved under prolonged stress may not be sustainable if underlying mental health concerns are not addressed. Poor mental health has been associated with reduced job satisfaction, strained workplace relationships, and diminished work performance.

The pandemic also introduced significant non-work stressors, such as ongoing concerns about health risks and childcare disruptions, which continue to affect employees even as schools have reopened (Su et al., 2022). While these external stressors contribute to employee strain, the present study focuses specifically on workplace-related factors, particularly employees’ perceptions of mattering at work, as a potential protective resource.

Grounded in mattering theory, this study examines whether feeling valued and significant within the workplace is associated with better employee well-being and performance outcomes, above and beyond general mental health challenges that may originate outside the work environment. By distinguishing workplace mattering from broader pandemic-related stressors, this study aims to clarify the role organizations play in supporting employee mental health and sustaining productivity in the post-pandemic context.

As we transition from working from home to returning to the office, it is the right time to address workplace mental health issues. The COVID-19 pandemic introduced widespread changes to work structures and social interaction, alongside increased psychological stress. Given that these contextual factors may influence mattering, depressive symptoms, and social media use, the present study accounts for pandemic-related impact to more precisely isolate associations linked to work-from-home arrangements.

## Social Media Addiction

A growing body of research has documented associations between social media use and adverse mental health outcomes, including depression, anxiety, and stress (Keles et al., 2020; Twenge et al., 2017). While social media platforms are widely used across age groups, their relevance to employees lies in how online interactions may shape perceptions of social value, belonging, and recognition. Research suggests that social media environments can foster social comparison, reinforce negative narratives, and amplify feelings of exclusion or underappreciation, particularly when individuals experience low support in their offline roles (Wood et al., 2016).

In the workplace context, employees who feel undervalued may turn to social media as an outlet for disengagement or expression, which can contribute to distraction, emotional exhaustion, and reduced productivity (Zivnuska et al., 2019). Rather than conceptualizing social media use as an addictive behavior, the present study views it as a contextual factor that may interact with employees’ perceptions of mattering at work. Examining this relationship helps clarify how external digital environments may reinforce or exacerbate workplace-related psychological strain.

In addition to examining mattering-related constructs, it is important to account for the broader pandemic-related stressors present during the period of data collection. The COVID-19 pandemic disrupted work routines, intensified uncertainty, and increased reliance on remote work and digital communication, all of which may influence employees’ perceptions of being valued, their psychological well-being, and their work experiences. Individuals who experienced COVID-19 infection may have faced additional stressors, including health-related anxiety, recovery demands, and social disruption, which could further shape perceptions of mattering and job satisfaction. Accordingly, the present study considered pandemic impact as a contextual factor and explored differences in mattering-related outcomes based on personal COVID-19 infection history.

## Methods and Procedures

**Participants:** A cross-sectional study was conducted between June 2022 and June 2023. Purposive, snowballing, and networking sampling were used. Participants were 60 adults working primarily from home who had at least one school-age child. These criteria were used to recruit individuals likely to have experienced altered work–life conditions during the pandemic, rather than to examine caregiving responsibilities as a study variable.

**Measures:** An electronic questionnaire was used. It consists of six sections:

- Mattering Scales: The participants completed four self-report subscales to measure the concept of mattering:
  1. the General Mattering Scale (GMS). It has five positively worded items and is designed to measure an individual's perceived significance and importance to others. Possible scores on the scale range from 5 to 20, with higher scores indicating a greater perception of mattering (Marcus & Rosenberg, 1987).
  2. Anti-Mattering Scale (AMS). It is a five-item self-report scale designed to measure an individual's perception of their insignificance, invisibility to others, or unimportance to others. Participants indicate their level of agreement with each item (Liu et al., 2023).
  3. Fear of Not Mattering Inventory (FNMI). The FNMI is a 5-item scale with response options ranging from “0” to “3.” The FNMI is articulated in Flett et al. (2025), where psychometric support is summarized (Cao et al., 2025). This scale has items such as, “How often do you worry that others will see you as unimportant or insignificant?” and “How often Do you worry that others will stop taking an interest in you?”
  4. Work Mattering Scale (WMS). The Work Mattering Scale (WMS; Jung & Heppner, 2017) is a 10-item self-report measure with five items that tap work that is high in societal mattering and five items that tap interpersonal mattering to others. Items are responded to with a 6-point Likert scale ranging from “Disagree very much (1)” to “Agree very much (6)” with higher summed scores reflecting greater mattering.
  Each measure comprises 5 to 10 scale items, making it relatively brief. The higher the score, the greater the significance, whether it is about mattering, anti-mattering, or the fear of not mattering (Liu et al., 2023).
- The World Health Organization Well-Being Index (WHO-5) is a brief self-report measure designed to assess current psychological well-being. The scale focuses on positive aspects of mental health, including mood, vitality, and interest in daily activities. Although the WHO-5 has been widely used as a screening tool for depressive symptoms in clinical and research settings, it was employed in the present study as an indicator of overall well-being and adjustment. Prior research has demonstrated strong construct validity and reliability for the WHO-5 as a unidimensional measure across diverse age groups (Topp et al., 2015).
- The Affective Job Satisfaction Scale: a four-item Brief Index of Affective Job Satisfaction (AJS) developed by Thompson and Phua in 2012, focuses primarily on emotions, requires minimal cognitive effort, and is concise. The scale has been thoroughly validated for internal consistency and stability over time, and its correlation with other relevant measures. Additionally, it is consistent across different nationalities, job levels, and types.
- The Center for Epidemiologic Studies Depression scale: This 10-item scale demonstrates high internal consistency and test-retest reliability. It also shows high convergent and divergent validities (Miller et al., 2008).
- The Bergen Social Media Addiction Scale: This scale is reliable for measuring social media addiction over extended periods. Its psychometric stability enhances its utility for longitudinal studies, making it a valuable tool for tracking changes in social media addiction behaviours (Gomez et al., 2024).
- Demographic data questionnaire: This explores possible group differences in levels of stress among people with varying genders, working conditions, and personal living conditions. Participants’ COVID-19 history (e.g., personal experience, family member's experience, etc.) was also collected.

### Procedures

Ethical approval for this study was obtained from the York University Research Ethics Committee (Certificate No. e2022-192). Participants provided online consent and completed the instruments. As a token of appreciation for their time, they received a $15 gift card. The data was analyzed using SPSS version 29.0.

## Results

Sixty questionnaires were distributed, and all 60 were returned, resulting in a 100% response rate. The mean age of the participants was 42.95 ± 6.25 years, with an age range of 31 to 56. Most participants (73.33%) were female and married (91.67%). Additionally, 91.67% of the participants held a degree higher than a bachelor's. Most participants were religious believers, with Buddhists accounting for 33.33% and Taoists for 35%. Many had a family income between $100,000 and $150,000 (76.67%). Moreover, 83.33% of the fifty couples had at least one child. Fifteen participants had been infected with COVID-19, and 27 had at least one family member contracted with COVID-19. The participants were employed in white-collar occupations, typically involving office-based work and professional roles, such as positions in information technology.

Means and Standard deviations of the significant study variables, as well as the reliability data for each scale, are listed in Table 1. When analyzing the effects of mattering theory on the study variables using Pearson correlations, it was observed that General Mattering Scale (GMS) scores had positive relationships with the scores of the Work Mattering Scale (WMS, *p* < .05) and the Affective Job Satisfaction Scale (AJS, *p* < .05). Anti Mattering Scale (AMS) scores showed negative relationships with scores of the Work Mattering Scale (*p* < .01), the Well-being Scale (*p* < .01), and the Affective Job Satisfaction Scale (*p* < .001). Social Media Addiction scores showed positive relationships with the Non-Mattering Scale scores (*p* < .05) and the Depression Scale (*p* < .01). Well-being was found to have positive relationships with the Work Mattering scale (*p* < .001) and the Affective Job Satisfaction (*p* < .01).

**Table 1.** Means, Standard Deviations (SD) and Reliabilities of Major Study Variables.

Variables	Mean	SD	Reliability (α)
GMS	15.62	3.15	.90
AMS	8.87	3.08	.86
NMS	4.64	4.14	.94
WMS	41.33	8.60	.89
WB	15.85	4.25	.86
AJS	14.54	3.06	.94
Depression	17.33	5.48	.73
Addiction	5.92	1.99	.70

GMS: General Mattering Scale, AMS: Anti-Mattering Scale, NMS: Non-Mattering Scale, WMS: Work Mattering Scale, WB: Well-being, AJS: Affective Job Satisfaction

There was no gender difference in the above variables. When using t-tests to compare individuals with and without COVID-19 infection on the study variables, significant differences in means were found in the General Mattering Scale (*p* < .05), Anti Mattering Scale (*p* < .01), Work Mattering Scale (*p* < .01), and Affective Job Satisfaction (*p* < .01). As can be seen in Tables 2 and 3, those participants who had become infected tended to report substantially higher levels of anti-mattering and lower levels of mattering, work mattering, and job satisfaction.

**Table 2.** Correlations (r) Among Variables.

Variables	GMS	AMS	FNMI	WMS	WB	AJS	Depression	Addiction
GMS	1							
AMS	−.52**	1						
FNMI	−.06	.38*	1					
WMS	.40*	−.42**	−.16	1				
WB	.29	−.47**	−.12	.53**	1			
AJS	.40*	−.62**	−.20	.28	.49**	1		
Depression	.21	.21	.28	.00	−.26	−.23	1	
Addiction	.18	.18	.32*	.01	−.19	.05	.41**	1

GMS: General Mattering Scale, AMS: Anti-Mattering Scale, FNMI: Fear of Not Mattering Inventory, WMS: Work Mattering Scale, WB: Well-being, AJS: Affective Job Satisfaction. ** < .01, * < .05

**Table 3.** Mean Differences in GMS, AMS, WM, and AJS Between With and Without COVID-19 Infection.

	With Infection	Without Infection	t Values	p Values
GMS	9.10	11.13	−1.82	.04*
AMS	5.80	3.21	2.44	.01*
WMS	35.00	43.51	−2.96	.00**
AJS	12.60	15.21	−2.47	.00**

GMS: General Mattering Scale, AMS: Anti-Mattering Scale, WMS: Work Mattering Scale, AJS: Affective Job Satisfaction. ** < .01, * < .05

## Discussion

Reece et al. (2021) suggested that individuals who feel important are likely to be more motivated and perform effectively. In our study, we found that participants who generally feel valued, as indicated by the General Mattering Scale (GMS), also tend to feel valued in their work environments, as indicated by the Work Matter Scale (WMS). This suggests that feeling valued generally extends to specific contexts, such as the workplace. When individuals feel personally appreciated, they are more likely to perceive themselves as necessary and valued in their professional roles (Reece et al., 2021).

Our results showed that, uniquely, in terms of the degree of associations between job satisfaction and the mattering facets, it was somewhat surprising that General mattering and anti-mattering were stronger predictors of job satisfaction, whereas work mattering was not significantly associated with job satisfaction. The most robust association was between higher levels of anti-mattering and lower levels of job satisfaction. The positive correlation between the General Mattering Scale (GMS) and the Affective Job Satisfaction Scale (AJS) suggests that feeling valued and important in general is linked to higher job satisfaction. This finding aligns with Reece et al.'s conclusion that employees believe they are significant to their organizations, especially when this belief is tied to their contributions (Reece et al., 2021). It is also consistent with Flett's suggestion that mattering can predict outcomes related to job satisfaction (Flett, 2022). Affective job satisfaction refers to an individual's emotional response to their job, encompassing feelings of pleasure or contentment derived from their job role. Thus, individuals who feel valued by others are more likely to have positive emotions about their jobs, indicating that the general perception of mattering can enhance job satisfaction (Flett, 2022).

Contrary to expectations, the study revealed no correlation between the General Mattering Scale (GMS) and the Anti-Mattering Scale (AMS). However, the Anti-Mattering Scale (AMS) showed negative relationships with three other scales: the Work Mattering Scale, the Well-being Scale, and the Affective Job Satisfaction Scale. This suggests that feelings of “anti-mattering” (the sense that one is insignificant or does not matter) are inversely related to positive outcomes in work, well-being, and job satisfaction. Participants who felt they did not matter were less likely to find their work meaningful or important. This feeling of insignificance affects their overall mental and emotional health and job satisfaction (Liu et al., 2023).

The total GMS score is 20. The mean GMS score of the study participants was 15.62 ± 3.15 (70.8%), indicating a high perceived significance to others. This was reflected in the AMS score, which was relatively low at 3.87 ± 3.08, suggesting that most participants did not feel insignificant to others. Further investigation is needed because studies showed a negative relationship between these two (Flett, 2022).

Regarding the measure of social media addiction, the chief finding that emerged was that higher reported levels of the fear of not mattering to others were associated with problematic social media use. This was the only mattering facet links with social media addiction scores. This finding is unique in that previous studies examined much younger participants and found that both anti-mattering and fear of not mattering were associated with greater social media addiction (Chen et al., 2025; Ding et al., 2024). Our sample of older participants tended to be more engaged with social media if they were characterized by concerns about being or becoming someone unimportant to others. It will be revealing to see if this finding would be replicated if these variables were assessed in a sample of older participants who were evaluated before, during, and after retirement.

The positive correlation between Social Media Addiction scores and both FNMI scores and Depression Scale scores in the present study suggests an interplay among social media usage, perceived self-worth, and mental health. Research has shown that billions of people use social networking websites such as Facebook, X, and Instagram, which can have adverse effects on mental health. Studies indicate that excessive use of social media can lead to anxiety, depression, and other challenges. People who limit their time on social media tend to be happier, while excessive use can trigger negative emotions and worsen symptoms of depression (Nittle, 2023).

Our findings align with Wood et al.'s study (2016), which also identified a positive correlation between social media usage and depression. While virtual interaction on social media does not provide the same psychological benefits as face-to-face contact, it still has some advantages when used appropriately. It can help people stay connected with family and friends worldwide, find new friends and communities, and network with people with similar interests or ambitions. However, several studies have found a strong link between heavy social media use and an increased risk for depression, anxiety, loneliness, self-harm, and even suicidal thoughts (Robinson & Smith, 2024). Further research could explore the causal pathways between social media addiction and depression. People who feel insignificant may seek validation and acknowledgment through social media. They may find satisfaction in receiving likes, comments, and shares, as these provide immediate feedback. Some people use social media to escape their feelings of insignificance by immersing themselves in an online persona or community where they feel valued (Wood et al., 2016). However, excessive use of social media for validation can become addictive. The temporary sense of mattering from online interactions reinforces the usage, even as the underlying feelings of insignificance persist or worsen. The positive correlation found between Social Media Addiction (SMA) scores and the Fear of Not Mattering to Others (FNMI)/Depression Scale scores in this study is consistent with the findings of Çimke and Cerit (2021). Their report emphasizes a complex relationship in which individuals with low perceived self-worth and depression often turn to social media for validation and an escape. Consistent with our findings, individuals who already experience low self-worth or depressive symptoms may be particularly vulnerable in work-from-home (WFH) arrangements. Working remotely can magnify feelings of insignificance, leading social media use to function as a coping strategy rather than merely a form of entertainment. WFH environments often limit spontaneous social cues—such as immediate feedback and visible acknowledgment of one's contributions—which are important sources of perceived mattering in traditional workplace settings. In the absence of these cues, individuals may increasingly rely on social media for validation. However, excessive reliance on social media can reinforce addictive usage patterns and further exacerbate fears of not mattering and depressive symptoms. These findings highlight the need for interventions that address both social media use and underlying psychological vulnerabilities, such as cognitive-behavioural strategies aimed at reducing fear of not mattering and maladaptive self-beliefs.

The positive relationships between well-being, the Work Mattering Scale, and Affective Job Satisfaction can be explained for two reasons. The Work Mattering Scale measures the extent to which individuals believe their work is significant (Liu et al., 2023). When employees feel that their work matters, it fosters a stronger sense of purpose and fulfillment, which enhances their overall well-being. A meaningful work experience can reduce stress and promote a positive emotional state, strengthening the connection between well-being and the feeling that one's work is important.

Affective Job Satisfaction reflects the emotional component of job satisfaction, focusing on how much employees enjoy their jobs. When people are emotionally satisfied with their work, they tend to experience positive emotions, such as happiness and contentment, which directly contribute to their psychological well-being (Thompson & Phua, 2012).

COVID-19 impact was measured to account for pandemic-related stressors that could influence work-from-home experiences, perceived mattering, mental health, and social media use during the data collection period. The significant differences observed between individuals with and without COVID-19 infection in the General Mattering Scale, Anti-Mattering Scale, Work Mattering Scale, and Affective Job Satisfaction may be attributed to various factors related to their experiences of illness and its impact on both personal and professional life. Participants who contracted COVID-19 may have faced increased stress, anxiety, or depressive symptoms, which could affect their sense of matter. The illness might have made them feel more isolated or undervalued, leading to lower scores on the General Mattering Scale and higher scores on the Anti-Mattering Scale. COVID-19 often necessitates quarantine, limiting social interactions and support from colleagues, friends, and family. This decreased connection can diminish feelings of importance and belonging, which is reflected in lower scores for work mattering and general mattering. Furthermore, many individuals who recover from COVID-19 report lingering symptoms such as fatigue and brain fog. These ongoing issues can reduce productivity and engagement at work, potentially resulting in lower job satisfaction and a diminished sense of mattering in the workplace. While there are many possible explanations, it is clear from the current results that being exposed to COVID-19 did seem to have some impact and in ways that impacted perceived lack of significance to other people. One overarching possibility is that whatever limitations were brought about illness resulted in less opportunity to engage with people in ways that left people feeling less valued to and by others.

The current study had some noteworthy limitations. First, the findings are derived from a unique sample but one that was relatively small. This could have impacted the findings in various respects. For instance, none of the mattering variables were associated with depression at conventional levels of statistical significance, which is inconsistent with a recent meta-analysis comparing mattering and anti-mattering in terms of their links with depression (Tonini et al., 2025). Second, the current results may not be generalizable, especially when it was considered that our participants tended to be from families that are relatively affluent relative to provincial norms of the average family salaries in Canada. Thus, the generalizability of these results needs to be evaluated rather than presumed.

## Conclusion

The study's findings emphasize the crucial role that mattering plays in employee well-being and job satisfaction. Furthermore, the relationship between social media addiction and employee depression, along with the considerable impact of COVID-19 on both employee mattering and job satisfaction, highlights the importance of these factors in the workplace. Understanding these issues can enable professionals to make informed decisions and implement effective strategies.
